# Metformin inhibits the proliferation of A431 cells by modulating the PI3K/Akt signaling pathway

**DOI:** 10.3892/etm.2015.2220

**Published:** 2015-01-27

**Authors:** YINGSHAN LIU, YAN ZHANG, KUN JIA, YUHAO DONG, WEIYUAN MA

**Affiliations:** 1 School of Medicine, Qilu Hospital, Shandong University, Jinan, Shandong 250012, P.R. China; 2 Department of Dermatology, Qilu Hospital, Shandong University, Jinan, Shandong 250012, P.R. China

**Keywords:** squamous cell carcinoma, skin, metformin, proliferation, Akt, PI3K

## Abstract

The ability of metformin, an antidiabetic drug with wide applications, to inhibit tumor cell growth has recently been discovered. The PI3K/Akt signaling pathway has been found to play an important role in the survival, proliferation and apoptosis of tumor cells. The aim of the present study was to explore the effect of metformin on the proliferation of A431 human squamous cell carcinoma cells and the underlying molecular mechanisms. A431 cells in the logarithmic growth phase were treated with 0, 15, 30, 45 and 60 mM metformin for 12, 24 and 36 h, respectively. Cell morphology with 45 mM metformin treatment for 24 h was observed under a microscope. The proliferation of A431 cells was detected by the Cell Counting kit-8 colorimetric method. The mRNA expression levels of PI3K and Akt were detected by reverse transcription-polymerase chain reaction (RT-PCR). The protein expression levels of PI3K, Akt and phosphorylated (p)-Akt were detected by western blot analysis. Metformin treatment caused morphological change in A431 cells and inhibited their proliferation in a significant time- and dose-dependent manner. RT-PCR results showed that the mRNA expression of PI3K was inhibited by metformin in a time- and dose-dependent manner (P<0.05). However, there was no significant change in the mRNA expression of Akt following metformin treatment (P>0.05). Western blotting results showed that the protein expression levels of PI3K and p-Akt were inhibited by metformin in a time- and dose-dependent manner (P<0.05). In conclusion, metformin significantly inhibited the proliferation of A431 cells in the current study, which may be strongly associated with the inhibition of the PI3K/Akt signaling pathway.

## Introduction

Cutaneous squamous cell carcinoma (SCC) originates in the epidermis or adnexal keratinocytes and has the highest incidence rate, next to basal cell carcinoma (BCC), of any non-melanocyte cell carcinoma (1). Due to its metastasis, the degree of malignancy and the risk of mortality for individuals with SCC is significantly higher than for those with BCC (2,3). A number of studies have indicated that ultraviolet (UV) light, ionizing radiation and chemical carcinogens play important roles in the development of SCC (1,4,5); however, its pathogenesis remains unknown. Currently, the main therapy for SCC is surgical excision (4,5). However, a carcinoma of a large size and the subsequently large excision in the affected area may severely affect the quality of life of the patient.

Metformin has been used in a wide range of applications for more than half a century. It is the first-line oral hypoglycemic agent for the treatment of type 2 diabetes and is recommended by the American Diabetes Association and the European Association for the Study of Diabetes (6). Previous studies have demonstrated that metformin is able to reduce the incidence rate of a variety of tumors in patients with diabetes (7–12). In *in vitro* studies, metformin has been found to reduce the volume of prostate tumors in nude mice (13) and inhibit the growth and proliferation of breast (14), prostate (13), cervical (15) and ovarian (16) cancer cells. However, the antitumor mechanism of metformin remains unclear. Akt, a serine/threonine protein kinase, has a vital function in multiple cellular processes including the regulation of cell growth, proliferation and apoptosis (17). PI3K is the most important upstream molecule to activate Akt (17). Previous studies have shown that the increased activities of PI3K and Akt play an important role in the development of numerous types of tumors (18).

In the present study, the A431 human squamous cell carcinoma cell line was cultured to investigate the effect of metformin on the proliferation of A431 cells and the underlying molecular mechanisms.

## Materials and methods

### Cells and reagents

SCC cell line A431 was purchased from the Cell Culture Center of the Chinese Academy of Sciences (Shanghai, China). Rabbit anti-human polyclonal antibodies against Akt (sc-8312), phosphorylated (p)-Akt (Ser 473; sc-135651) and PI3K (sc-423) were purchased from Santa Cruz Biotechnology (Dallas, CA, USA). The polyclonal rabbit anti-human antibody against β-actin was purchased from Sigma-Aldrich (SAB2100037; St. Louis, MO, USA). The secondary polyclonal goat anti-rabbit antibody labeled with horseradish peroxidase (HRP) was purchased from EarthOx LLC (E030120-01; San Francisco, CA, USA). Metformin hydrochloride with 98.8% purity was purchased from Shouguang Fukang Pharmaceutical Co., Ltd. (Shouguang, China) and was diluted with phosphate-buffered saline (PBS) into 1 M solution for storage. Dulbecco’s modified Eagle’s medium (DMEM), fetal bovine serum and 0.25% trypsin digestion solution were purchased from Gibco (Grand Island, NY, USA). TRIzol^®^ reagent was purchased from Invitrogen Life Technologies (Carlsbad, CA, USA). 2X Power Taq polymerase chain reaction (PCR) MasterMix and the Bioteke Super reverse transcription (RT)-kit were purchased from BioTeke Corporation (Beijing, China). RIPA lysis buffer and phenylmethanesulfonylfluoride (PMSF) were purchased from Beyotime Institute of Biotechnology (Jiangsu, China). The Cell Counting kit-8 (CCK-8) was purchased from Dojindo Molecular Technologies, Inc. (Kunamoto, Japan). The polyvinylidene fluoride (PVDF) membrane was purchased from the Pall Corporation (Port Washington, NY, USA). The study was approved by the Ethics Committee of Shandong University (Jinan, China).

### Cell culture and metformin treatment

A431 cells were cultured in DMEM containing 10% fetal bovine serum at 37°C in a 5% CO_2_ incubator. Following three stable passages, the A431 cells were seeded onto 6-well plates with a density of 5×10^4^ cells/well. After 24 h of inoculation, the culture medium was replaced with serum-free medium and different concentrations of metformin (0, 15, 30, 45 and 60 mM) were added for 12, 24 and 36 h. The control group received the corresponding volume of PBS.

### Cell morphology observation

The morphology of the A431 cells with the treatment of 45 mM metformin for 24 h was observed under an inverted microscope (Olympus CKX31; Olympus Corporation, Tokyo, Japan). These values were selected as they produced the most marked morphological results.

### Cell proliferation assay

A431 cells were inoculated into 96-well plates with a density of 5×10^4^ cells/well at 37°C in a 5% CO_2_ incubator for 24 h. Metformin was added to the wells with final concentrations of 0, 15, 30, 45 and 60 mM for an incubation period of 12, 24 and 36 h. A total of 10 μl CCK-8 solution was added to each well and the optical density (OD) values were measured at 450 nm using a quantitative automatic microplate reader (model no. 2010; Anthos Labtec Co., Ltd., Salzburg, Austria). The rate of cell growth inhibition (%) was calculated as follows: (OD control - OD metformin)/OD control ×100.

### Total RNA extraction and RT-PCR analysis

Total RNA was extracted according to the manufacturer’s instructions of the TRIzol^®^ reagent and RT was carried out using the RT-kit for the total RNA. For each sample, 1 μg total RNA, random primers and M-MuLV reverse transcriptase enzyme were added to the 20 μl RT reaction system. Complementary DNA (cDNA) was synthesized under the following conditions: 42°C for 50 min and then 70°C for 10 min. The primers were provided by the Sangon Biotech Co., Ltd. (Shanghai, China), and are shown in Table I. PCR was performed in a 50-μl reaction system including 5 μl cDNA, 2.5 μl upstream and downstream primer, 25 μl 2X PCR mix and 15 μl triple-distilled water. The PCR cycler used was the Icycling 96 Gradient PCR Instrument (BioTeke Corporation, Beijing, China). The amplification conditions of the PCR were as follows: 94°C for 5 min, followed by 33 cycles at 94°C for 30 sec, 55°C for 30 sec, 72°C for 30 sec, and finally 72°C for 10 min. The amplification products of PCR were electrophoresed in a 2% agarose gel stained with ethidium bromide. The optical density of each sample band was captured using a UV gel imaging system (Tanon-2500R; Tanon Science & Technology Co., Ltd, Shanghai, China) and analyzed using ImageJ 1.47v software (National Institutes of Health, Bethesda, MD, USA). The OD ratio of the target band to GAPDH was defined as the relative mRNA expression of the target band.

### Western blot analysis

RIPA protein lysis buffer containing 1 mM PMSF was used to lyse cells on ice and the protein concentration was determined using a bicinchoninic acid (BCA) assay kit (Beyotime Institute of Biotechnology, Shanghai, China). A total of 50 μg protein from every sample was loaded onto a 10% SDS-PAGE gel for constant voltage electrophoresis and transferred to a PVDF membrane under a constant 1-mA/cm^2^ current. The nonspecific binding sites on the PVDF membranes were blocked by Tris-buffered saline and Tween 20 (TBST) buffer containing 5% skimmed milk for 1 h. The PVDF membranes were subsequently incubated with primary antibodies (dilution, 1:1,000) at 4°C overnight followed by incubation with the corresponding secondary antibody labeled with HRP (dilution, 1:10,000) at room temperature for 2 h. The membranes were developed on film using enhanced chemiluminescence (ECL) and scanned using an electrophoresis gel imaging analysis system (Tanon-5000R; Tanon Science & Technology Co., Ltd). β-actin was used as the internal control. The relative absorbance ratios of target protein to β-actin were defined as the relative values of target protein.

### Statistical analyses

Quantitative data are expressed as the mean ± standard deviation. Statistical analyses were performed using the SPSS statistical software package, version 20.0 (IBM SPSS, Armonk, NY, USA). The Student’s t-test was used to compare the difference between two groups and P<0.05 was considered to indicate a statistically significant difference.

## Results

### Effect of metformin on A431 cell morphology

**Figure 1:**
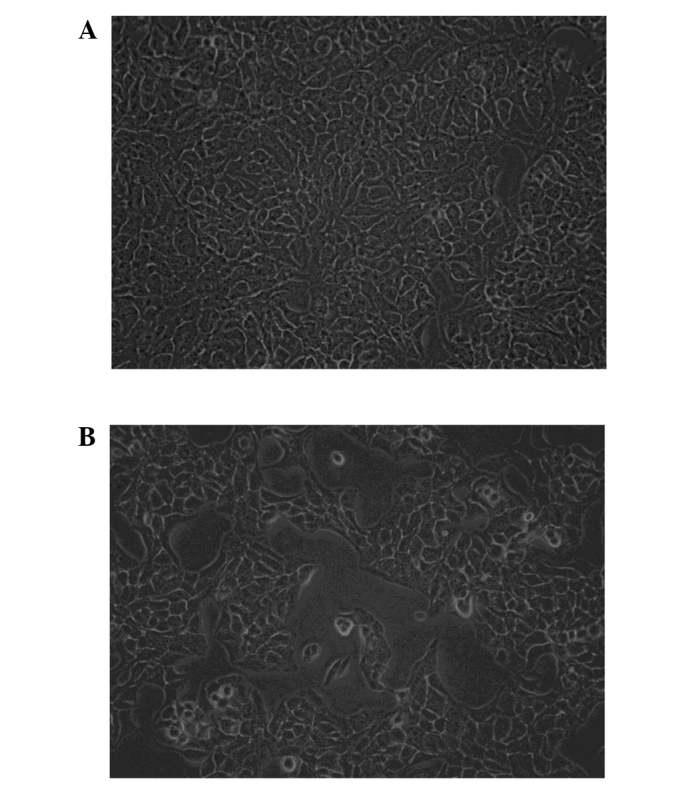
Morphological analysis of A431 cells following metformin treatment. Cells were observed under an inverted optical microscope (magnification, ×200). A431 cells (A) without metformin and (B) treated with 45 mM metformin for 24 h.

Following treatment with 45 mM metformin for 24 h, poor growth of the A431 cells with increasing karyopyknosis was observed. There were scattered areas with no cell growth and the number of floating cells was increased significantly in the treatment group compared with that in the untreated group. The A431 cells that were not subjected to metformin treatment were distributed evenly and were of a similar size (Fig. 1).

### Inhibitory effect of metformin on the proliferation of A431 cells

**Figure 2:**
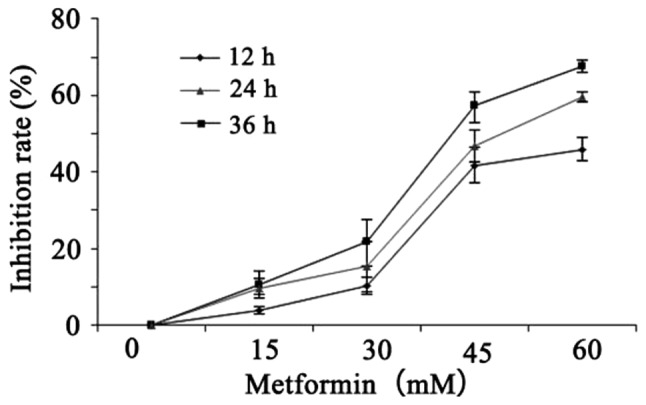
Inhibition of A431 cell proliferation by metformin with different concentrations and durations. At different concentrations of metformin, the inhibition rate of A431 cell proliferation increased with the treatment duration. For the same treatment duration, the inhibition rate of A431 cell proliferation increased with the concentration of metformin used. The optical density (OD) values were measured to assess the degree of cell proliferation. The inhibition rate = (OD control - OD metformin)/OD control ×100. Data are expressed as mean ± standard deviation and all experiments were repeated three times.

A431 cells were seeded onto 96-well plates. Following treatment with metformin at different concentrations (0, 15, 30, 45 and 60 mM) for 12, 24 and 36 h, cell activity was detected by the CCK-8 method. With the increase in treatment concentration and duration, the inhibitory effect of metformin on A431 cell proliferation gradually increased (Table II and Fig. 2). These results demonstrated that metformin inhibited the proliferation of A431 cells in a time- and dose-dependent manner.

### Effect of metformin on the mRNA expression levels of PI3K and Akt

**Figure 3:**
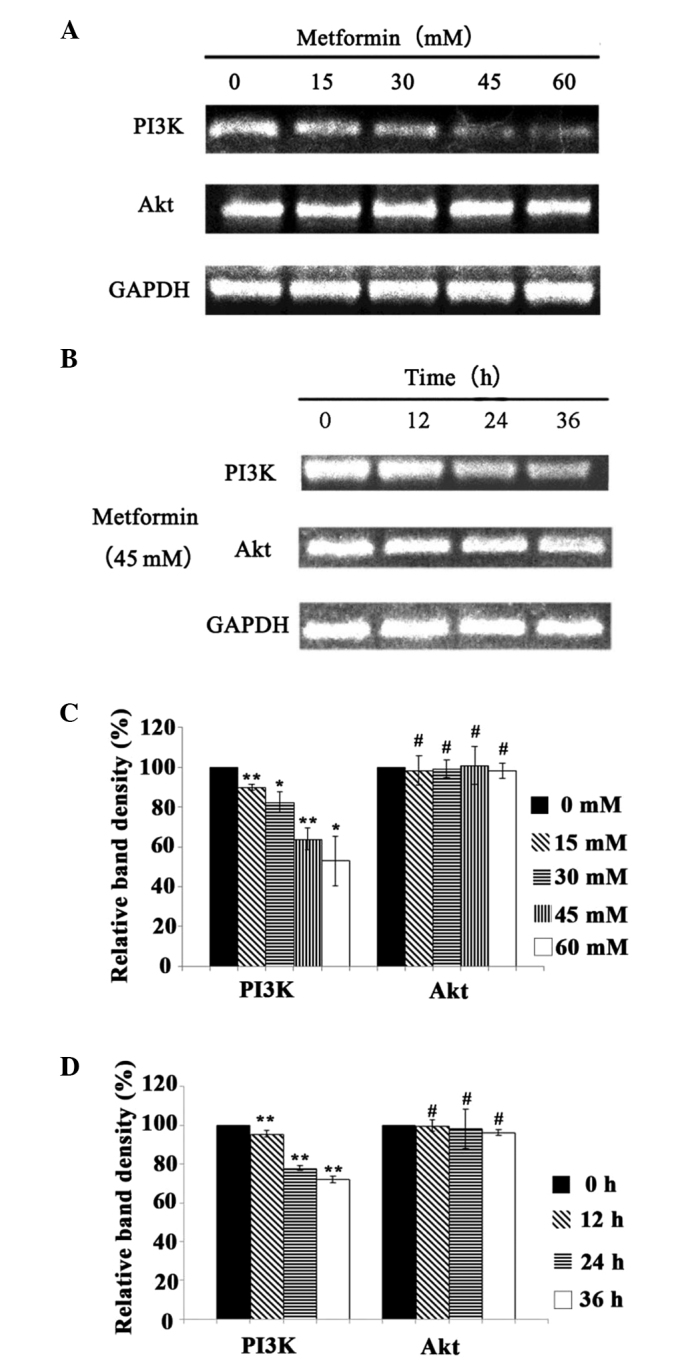
mRNA expression levels of PI3K and Akt in A431 cells following metformin treatment. The mRNA expression levels of PI3K and Akt in A431 cells treated with (A and C) 0, 15, 30, 45, 60 mM metformin for 24 h and (B and D) 45 mM metformin for 12, 24 and 48 h. The mRNA levels of PI3K and Akt were detected by reverse transcription-polymerase chain reaction. The relative optical density for mRNA was measured using Image J software, with GAPDH as an internal reference. All data, repeated by three independent experiments, are presented as mean ± standard deviation. ^*^P<0.05, ^**^P<0.01 and ^#^P>0.05 vs. control group.

Following treatment with metformin at different concentrations (0, 15, 30, 45 and 60 mM) for 24 h, the mRNA expression levels of PI3K and Akt were detected by RT-PCR (Fig. 3). In addition, following treatment with 45 mM metformin for 12, 24 and 36 h, the mRNA expression levels of PI3K and Akt were also detected by RT-PCR (Fig. 3). The mRNA expression level of PI3K decreased with the increase in the concentration and duration of metformin treatment (P<0.05), which indicated that metformin inhibited the mRNA expression of PI3K in a time- and concentration-dependent manner. Following treatment with metformin at different concentrations, the mRNA expression of Akt showed no significant difference compared with that of the control group (P>0.05). Furthermore, following treatment with 45 mM metformin for different durations, the mRNA expression levels of Akt also demonstrated no significant difference from that in the control (P>0.05). Thus, metformin did not have a significant effect on the gene expression of Akt.

### Effect of metformin on the protein expression levels of PI3K and p-Akt

**Figure 4:**
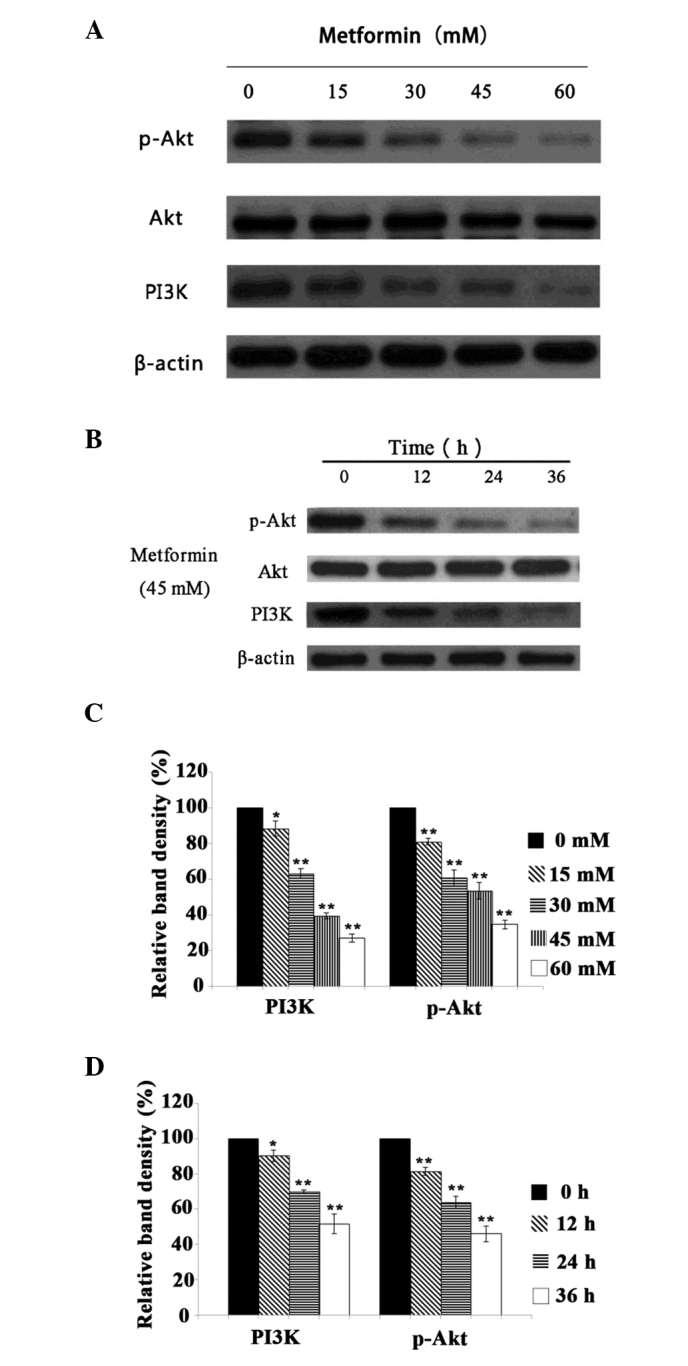
Protein expression levels of PI3K and phosphorylated-Akt in A431 cells following metformin treatment. Protein expression levels of PI3K and phosphorylated-Akt in A431 cells treated with (A and C) 0, 15, 30, 45, 60 mM metformin for 24 h and (B and D) 45 mM metformin for 0, 12, 24 and 36 h. The protein levels of PI3K and phosphorylated-Akt were detected by western blot analysis. The optical density of the protein was measured using Image J software, with β-actin as an internal reference. All data, repeated by three independent experiments, are presented as mean ± standard deviation. ^*^P<0.05 and ^**^P<0.01 vs. control group.

It is accepted that Akt plays a key role in the regulation of the growth and proliferation of tumor cells (17). The inhibition of Akt activity may suppress cancer cell growth and promote the apoptosis of these cells. The present study observed that treatment with metformin decreased the protein expression levels of PI3K and p-Akt in A431 cells in a time- and concentration-dependent manner (P<0.05; Fig. 4). These results indicate that the mechanism by which metformin inhibits the proliferation of A431 cells may involve modulation of the PI3K/Akt signaling pathway.

## Discussion

Metformin, the first-line drug for the treatment of type 2 diabetes, can reduce hepatic glucose output and increase glucose uptake by activating the AMP-activated protein kinase (AMPK) signaling pathway (19). Decensi *et al* performed a meta-analysis on 11 studies and found that compared with other antidiabetic drugs, metformin led to a 31% reduction in the total relative risk (RR) of cancer (RR, 0.69; 95% confidence interval, 0.61–0.79) (8). At present, studies investigating the inhibition of tumor cell proliferation by metformin remain focused on the AMPK signaling pathway (20).

Zakikhani *et al* reported that the inhibitory effect of metformin on the proliferation of the MCF-7 human breast cancer cell line decreased after the expression of the AMPK subunit was inhibited by siRNA (14). Yung *et al* (15) observed that the growth of cervical cancer cells was inhibited by metformin in a time- and dose-dependent manner. Similar results were obtained following treatment with AMPK activators including 5-amino-1-β-D-ribofuranosyl-imidazole-4-carboxamide (AICAR) and A23817, which further confirmed that the activation of AMPK was able to suppress cervical cancer cell growth. However, Ben Sahra *et al* demonstrated that the inhibitory effect of metformin on human prostate cancer cell proliferation did not decrease when the two catalytic subunits of AMPK were suppressed by siRNA. This difference in the response to AMPK suppression may have been due to the different sources of the cancer cells used and the incomplete inhibition of AMPK (13). Byekova *et al* found that compared with the expression of p-AMPK in normal keratinocytes and HaCaT cells, that in A431 cells was significantly higher and metformin was able to increase the expression of p-AMPK in a dose-dependent manner in HaCaT cells, but not in A431 cells (21).

In addition to the classic AMPK signaling pathway, the PI3K/Akt signaling pathway also plays an important role in the regulation of cell proliferation by metformin. Zakikhani *et al* reported that metformin inhibited the growth of breast cancer cells through the activation of AMPK and the inhibition of Akt to suppress certain regulatory molecules, including mTOR and S6 kinase (S6K) (14). Yung *et al* demonstrated that the activation of AMPK induced by 25 mM metformin caused the upregulation of p-AMPK expression and the downregulation of p-Akt expression, and the phosphorylation of FOXO3a and Forkhead Box M1 (FOXM1) in cervical cancer cell line C33A. The study also revealed that following the inhibition of FOXO3a by siRNA, the expression of FOXM1 was not significantly altered by metformin, which indicated that metformin may have suppressed cervical cancer cell growth through activation of the AMPK and inhibition of the Akt/FOXO3a/FOXM1 signaling pathways (15). Karnevi *et al* found that the antiproliferative effect of metformin on pancreatic cancer cells was associated with the activation of AMPK Thr172, which was able to suppress the phosphorylation of Akt and eventually inhibit cell proliferation (22).

The present study showed that metformin treatment induced morphological changes and inhibited the proliferation of A431 cells in a significant time- and dose-dependent manner. The mRNA expression level of PI3K and protein expression levels of PI3K and p-Akt were significantly decreased by metformin in a time- and dose-dependent manner. The mRNA expression of Akt did not alter with metformin treatment. Thus, it is speculated that metformin significantly inhibits phosphorylation in the PI3K/Akt signaling pathway, thereby suppressing A431 cell proliferation. The activation of Akt regulates cell cycle progression through multiple downstream pathways. The cyclin-dependent kinase inhibitors, p27kip1 on T157 and p21Clip/WAF1 on T145, are phosphorylated by Akt to prevent the localization of p27 and p21 to the nucleus, which attenuates their inhibitory cell-cycle effects (17). The stability of c-jun, c-myc and cyclins D and E, the important molecules in the G1 to S phase transition of the cell cycle, can be enhanced by Akt through the inhibition of GSK3, which leads to cell cycle arrest in the G1 phase (23,24). Akt activates eIF4E by inhibiting 4EBP1 through the mTORC1 pathway to enhance the mRNA expression levels of cyclin D1 and c-Myc (25). The DNA damage effector kinase Chk1 on serine 280 can be directly phosphorylated by Akt, thereby stimulating the movement of Chk1 into the cytoplasm which blocks its function and promotes cell cycle progression from the G2 to the M phase (26). However, the exact molecular mechanism of the inhibition of PI3K/Akt pathway by metformin and the resulting suppression of cell proliferation remains unclear and requires clarification by further study.

In summary, metformin inhibited the proliferation of A431 cells *in vitro* in a time- and dose-dependent manner, which was significantly associated with the phosphorylation level of the PI3K/Akt signaling pathway. With increasing attention devoted to the antitumor effect of metformin, the drug may lead to improved treatment options for patients with SCC and become a potential therapeutic treatment for SCC.

## Figures and Tables

**Table I:** Primer sequences for reverse transcription-polymerase chain reaction (RT-PCR).

Target genes	Primer sequence	Product size (bp)
PI3K	Forward: 5′-ACTTTGCGACAAGACTGC-3′ Reverse: 5′-GCCCTATCCTCCGATTAC -3′	337
Akt	Forward: 5′-ACAGCAAAGCAGGAGTATAAGA-3′ Reverse: 5′-CCAAACGAAACCAAGTCAA-3′	336
GAPDH	Forward: 5′-AAGTACTCCGTGTGGATCGG-3′ Reverse: 5′-ATGCTATCACCTCCCCTGTG-3′	360

**Table II:** Inhibition of A431 cell proliferation by metformin treatment with different concentrations and durations.

Time	Untreated (%)	15 mM (%)	30 mM (%)	45 mM (%)	60 mM(%)
12 h	0	0.0388±0.0087	0.1030±0.0225	0.4181±0.0470	0.4593±0.0297
24 h	0	0.1051±0.0349	0.2192±0.0561	0.5723±0.0375	0.6748±0.0179
36 h	0	0.0964±0.0262	0.1527±0.0655	0.4678±0.0428	0.5962±0.0137
