# Metformin inhibits the proliferation of A431 cells by modulating the PI3K/Akt signaling pathway

**DOI:** 10.3892/etm.2022.11372

**Published:** 2022-05-16

**Authors:** Yingshan Liu, Yan Zhang, Kun Jia, Yuhao Dong, Weiyuan Ma

Exp Ther Med 9:1401–1406, 2015; DOI: 10.3892/etm.2015.2220

Subsequently to the publication of the above article, an interested reader drew to the authors’ attention that the mRNA bands presented in Fig. 3B appeared to contain an overlapping band held in common, comparing between the Akt and the GAPDH bands. The authors realized that an error had indeed been made during the assembly of the data into this figure; essentially, the incorrect bands had been included in the figure to represent the Akt mRNA expression experiment.

In view of the time that has elapsed since this article was published, the authors elected to repeat the agarose gel electrophoresis experiments in question, and have found that the new data supports the same conclusions as those presented in the original paper. The revised version of Fig. 3 is shown opposite, now featuring new data for Fig. 3B, showing the mRNA expression levels of PI3K and Akt in A431 cells following treatment with 45 mM metformin for 12, 24 and 36 h.

Note that this error did not have a major impact on either the overall results or on the conclusions reported in this study. The authors regret the errors that were made during the compilation of the data into Fig. 3B. All the authors agree with the publication of this corrigendum, and are grateful to the Editor of *Experimental and Therapeutic Medicine* for granting them the opportunity to publish this; furthermore, they apologize to the readership for any inconvenience caused.

## Figures and Tables

**Figure 3:**
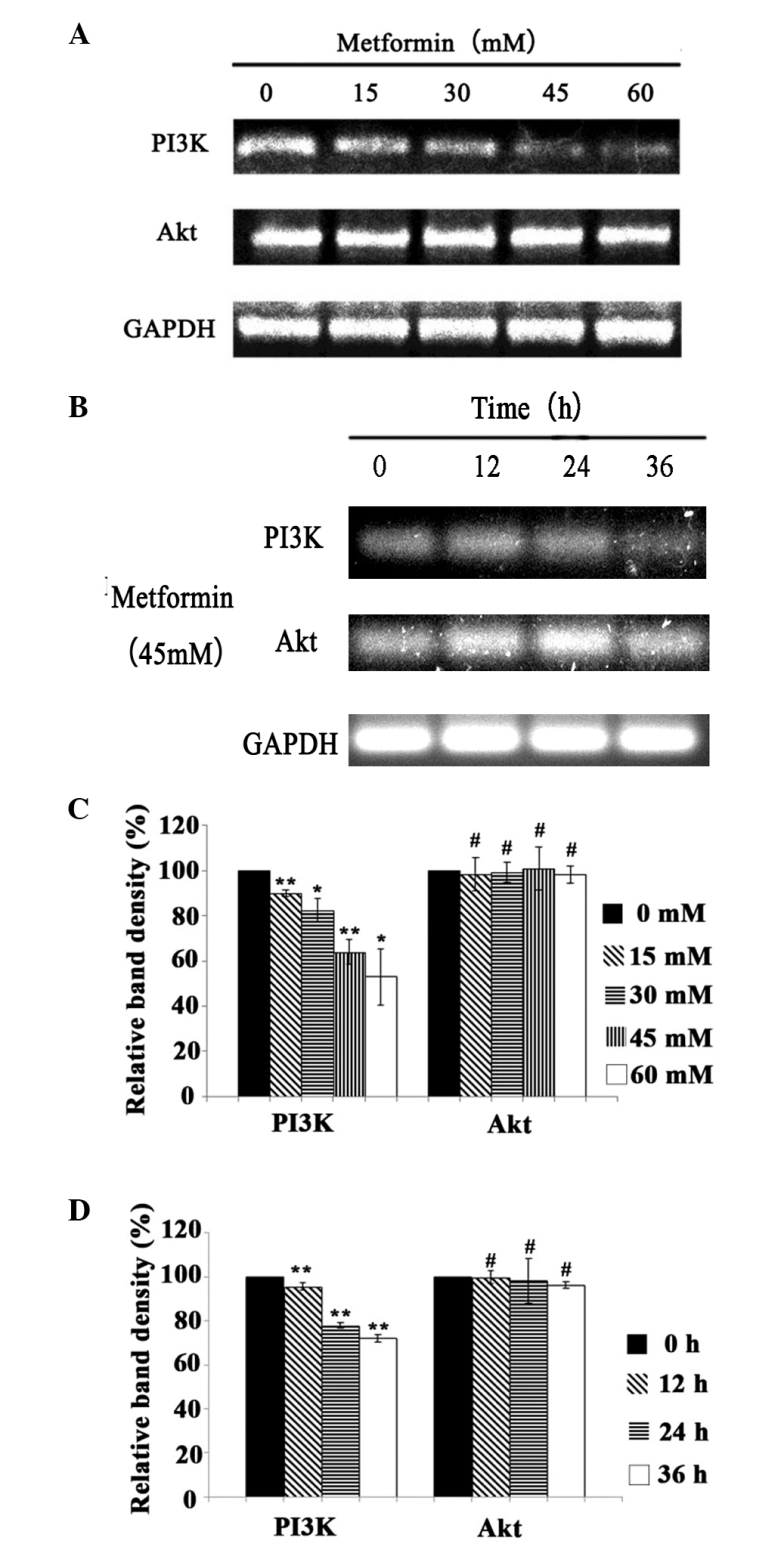
mRNA expression levels of PI3K and Akt in A431 cells following metformin treatment. The mRNA expression levels of PI3K and Akt in A431 cells treated with (A and C) 0, 15, 30, 45, 60 mM metformin for 24 h and (B and D) 45 mM metformin for 12, 24 and 36 h. The mRNA levels of PI3K and Akt were detected by reverse transcriptionpolymerase chain reaction. The relative optical density for mRNA was measured using Image J software, with GAPDH as an internal reference. All data, repeated by three independent experiments, are presented as mean ± standard deviation. ^*^P<0.05, ^**^P<0.01 and ^#^P>0.05 vs. control group.

